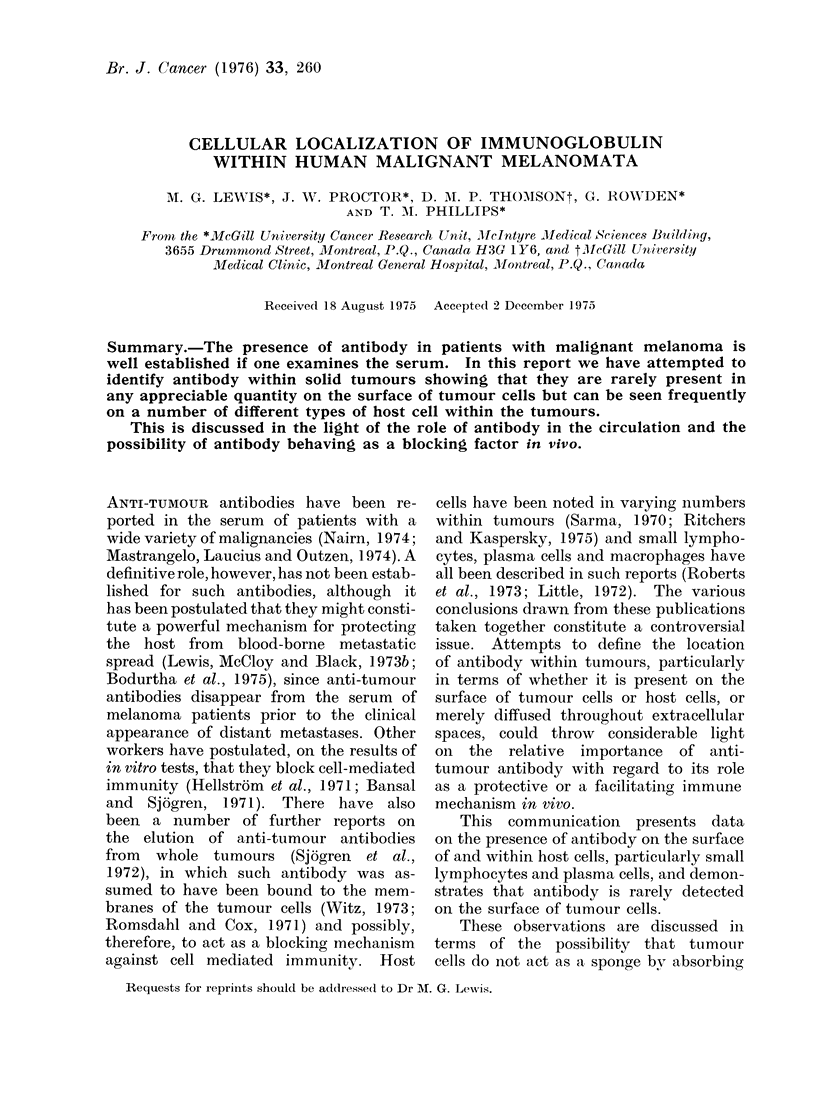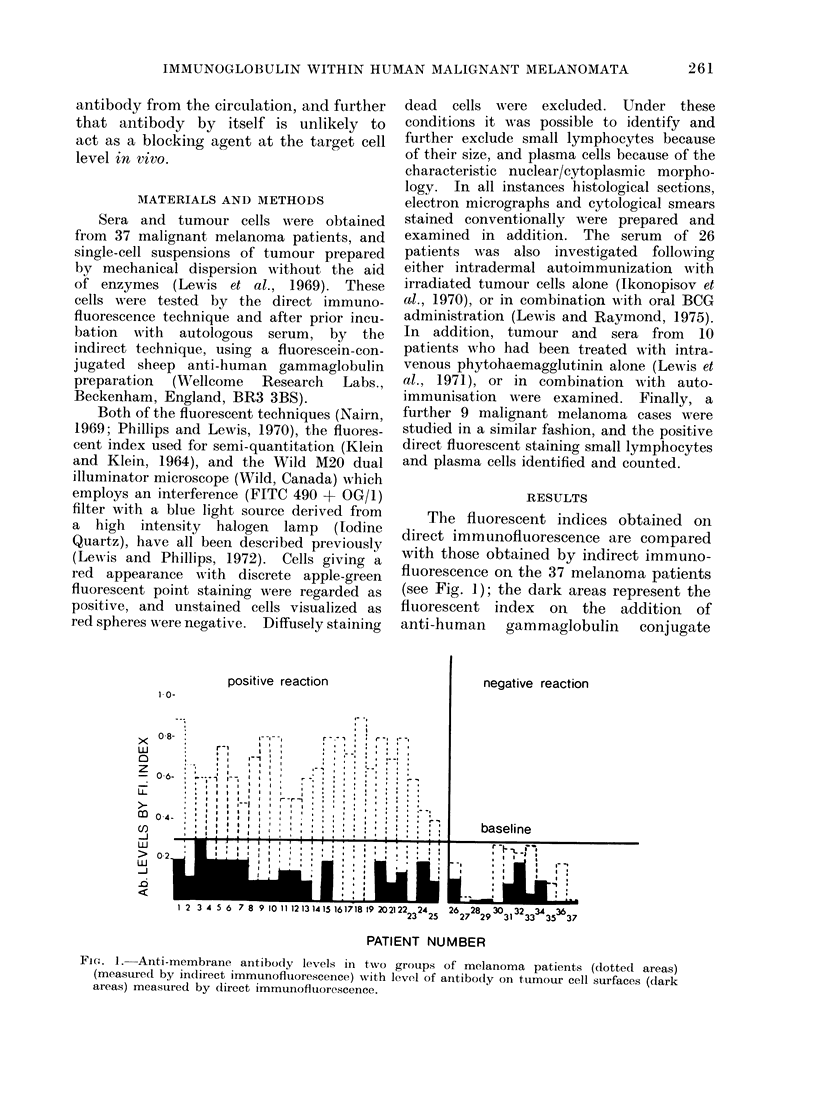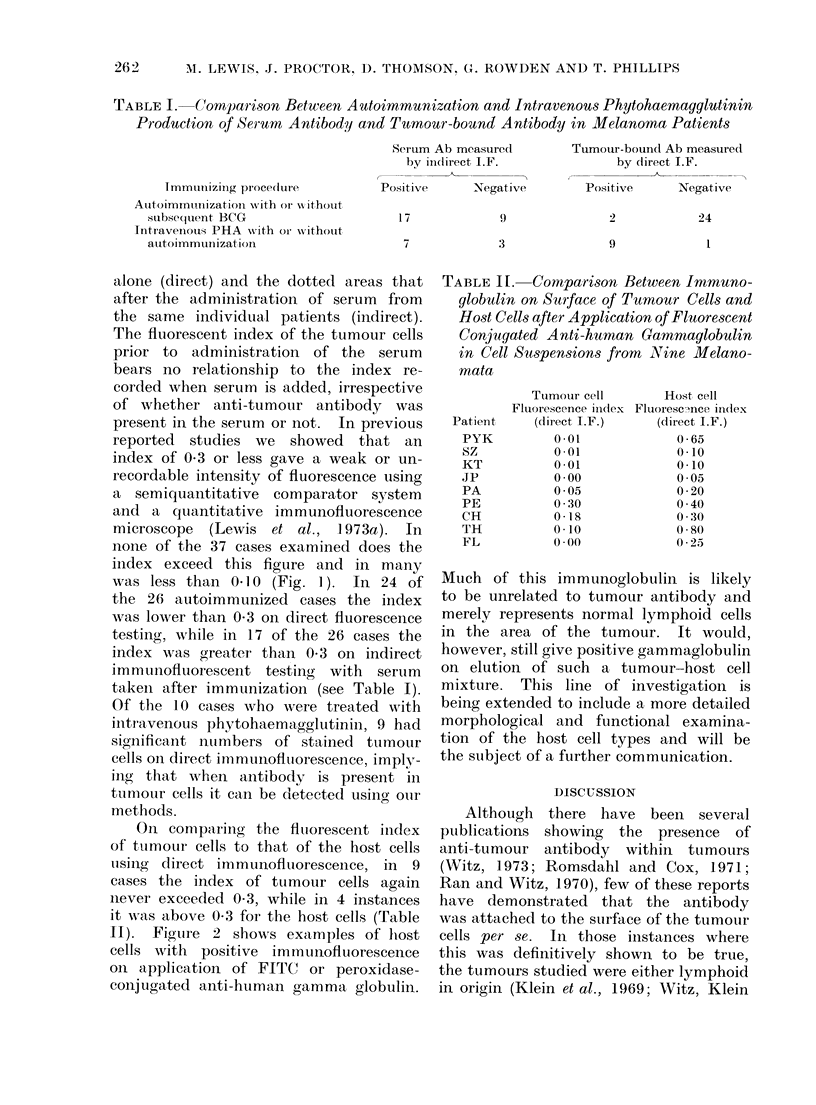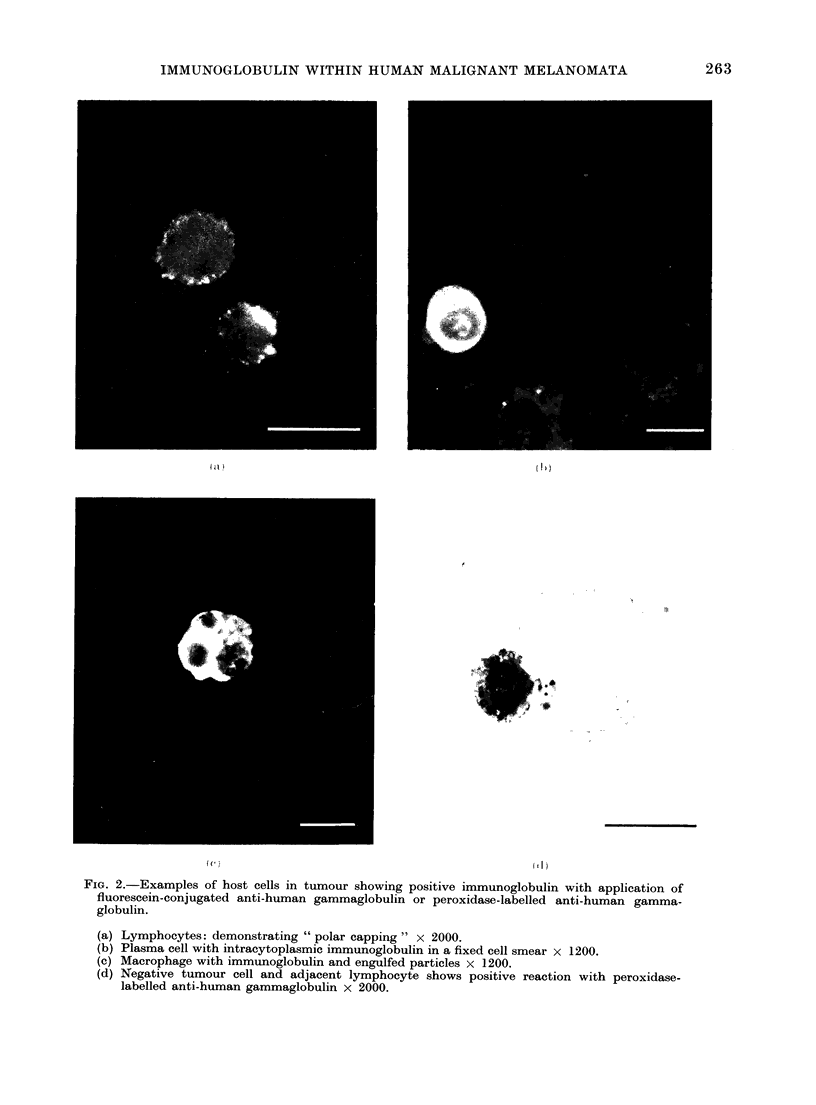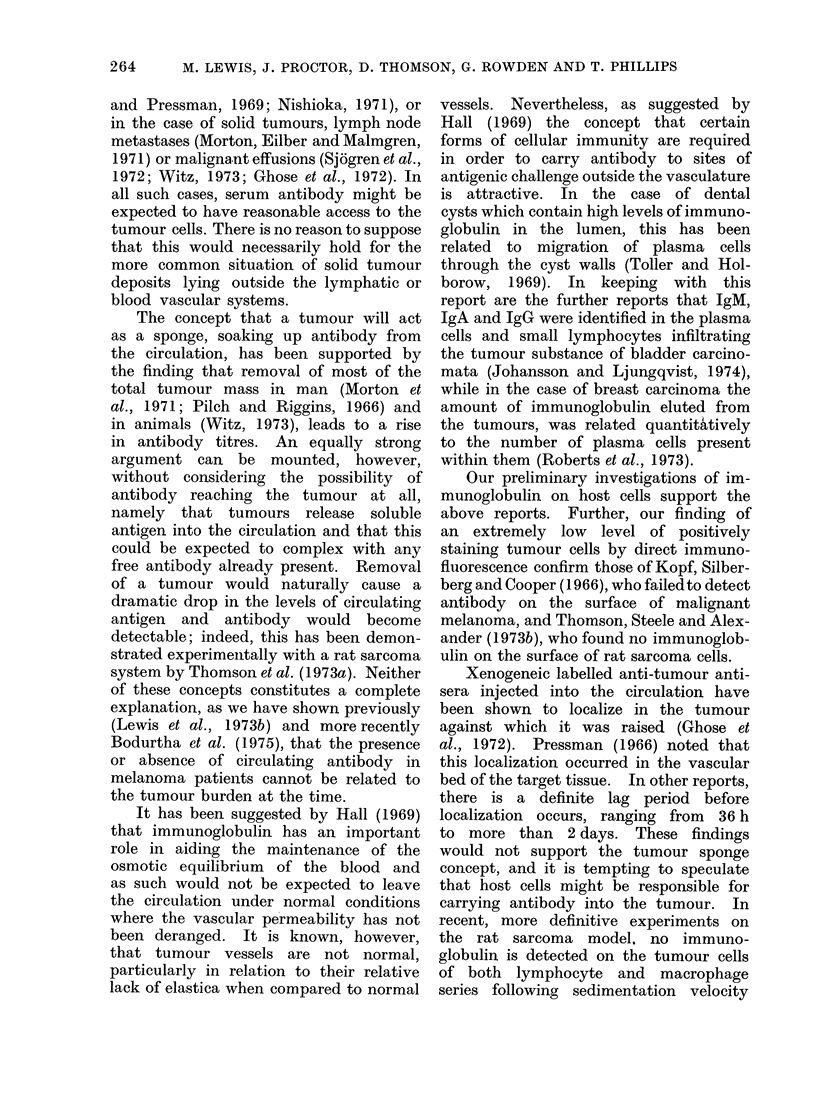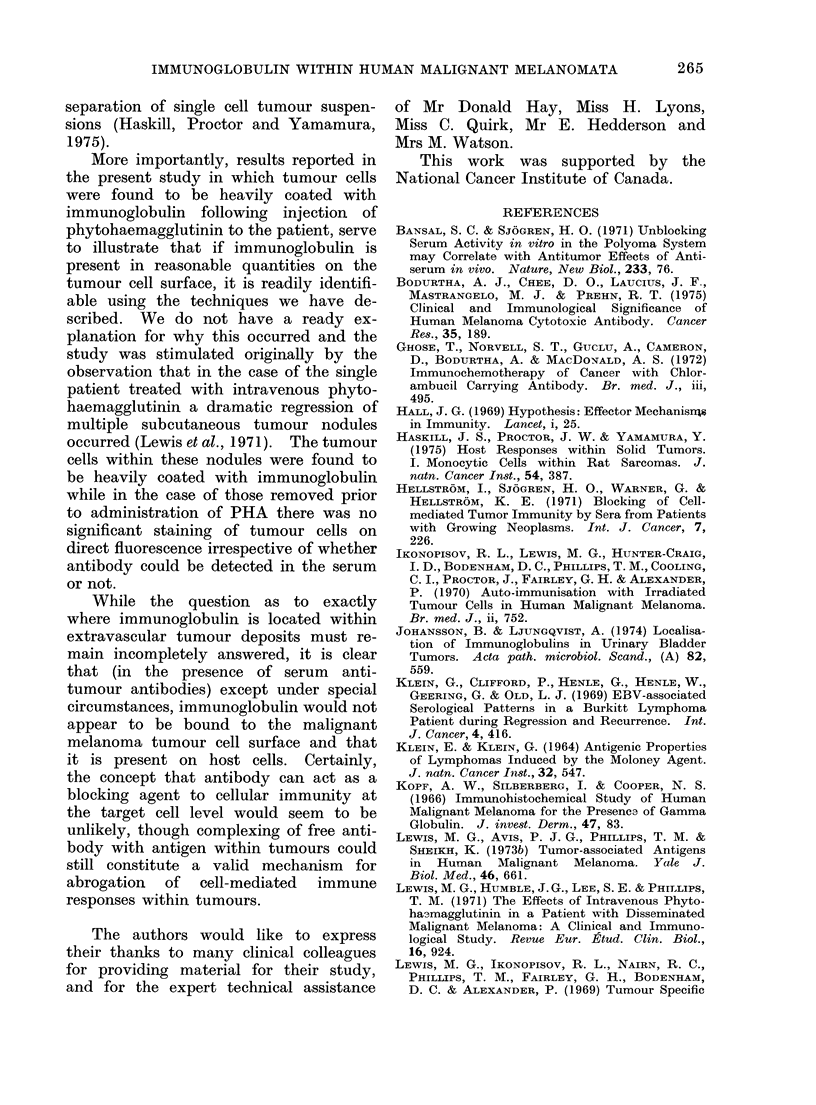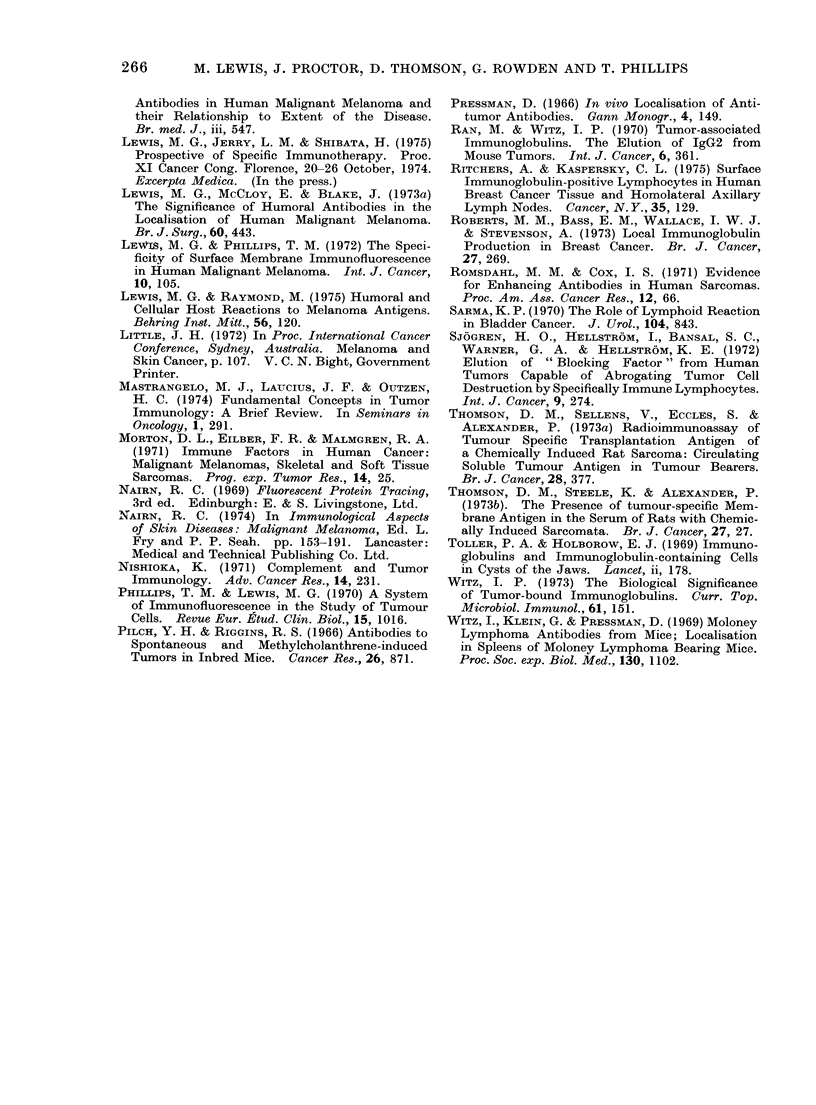# Cellular localization of immunoglobulin within human maglignant melanomata.

**DOI:** 10.1038/bjc.1976.38

**Published:** 1976-03

**Authors:** M. G. Lewis, J. W. Proctor, D. M. Thomson, G. Rowden, T. M. Phillips

## Abstract

**Images:**


*Br. J. Cancer* (1976) **33**, 260

### CELLULAR LOCALIZATION OF IMMUNOGLOBULIN WITHIN HUMAN MALIGNANT MELANOMATA

M. G. LEWIS*, J. W. PROCTOR*, D. M. P. THOMSON†, G. ROWDEN* AND T. M. PHILLIPS*

*From the *McGill University Cancer Research Unit, McIntyre Medical Sciences Building, 3655 Drummond Street, Montreal, P.Q., Canada H3G 1Y6, and †McGill University Medical Clinic, Montreal General Hospital, Montreal, P.Q., Canada*

Received 18 August 1975 Accepted 2 December 1975

**Summary.—The presence of antibody in patients with malignant melanoma is well established if one examines the serum. In this report we have attempted to identify antibody within solid tumours showing that they are rarely present in any appreciable quantity on the surface of tumour cells but can be seen frequently on a number of different types of host cell within the tumours.**

**This is discussed in the light of the role of antibody in the circulation and the possibility of antibody behaving as a blocking factor *in vivo*.**

ANTI-TUMOUR antibodies have been reported in the serum of patients with a wide variety of malignancies (Nairn, 1974; Mastrangelo, Laucius and Outzen, 1974). A definitive role, however, has not been established for such antibodies, although it has been postulated that they might constitute a powerful mechanism for protecting the host from blood-borne metastatic spread (Lewis, McCloy and Black, 1973*b*; Bodurtha *et al.*, 1975), since anti-tumour antibodies disappear from the serum of melanoma patients prior to the clinical appearance of distant metastases. Other workers have postulated, on the results of *in vitro* tests, that they block cell-mediated immunity (Hellström *et al.*, 1971; Bansal and Sjögren, 1971). There have also been a number of further reports on the elution of anti-tumour antibodies from whole tumours (Sjögren *et al.*, 1972), in which such antibody was assumed to have been bound to the membranes of the tumour cells (Witz, 1973; Romsdahl and Cox, 1971) and possibly, therefore, to act as a blocking mechanism against cell mediated immunity. Host cells have been noted in varying numbers within tumours (Sarma, 1970; Ritchers and Kaspersky, 1975) and small lymphocytes, plasma cells and macrophages have all been described in such reports (Roberts *et al.*, 1973; Little, 1972). The various conclusions drawn from these publications taken together constitute a controversial issue. Attempts to define the location of antibody within tumours, particularly in terms of whether it is present on the surface of tumour cells or host cells, or merely diffused throughout extracellular spaces, could throw considerable light on the relative importance of anti-tumour antibody with regard to its role as a protective or a facilitating immune mechanism *in vivo*.

This communication presents data on the presence of antibody on the surface of and within host cells, particularly small lymphocytes and plasma cells, and demonstrates that antibody is rarely detected on the surface of tumour cells.

These observations are discussed in terms of the possibility that tumour cells do not act as a sponge by absorbing

Requests for reprints should be addressed to Dr M. G. Lewis.

antibody from the circulation, and further that antibody by itself is unlikely to act as a blocking agent at the target cell level *in vivo*.

### MATERIALS AND METHODS

Sera and tumour cells were obtained from 37 malignant melanoma patients, and single-cell suspensions of tumour prepared by mechanical dispersion without the aid of enzymes (Lewis *et al.*, 1969). These cells were tested by the direct immunofluorescence technique and after prior incubation with autologous serum, by the indirect technique, using a fluorescein-conjugated sheep anti-human gammaglobulin preparation (Wellcome Research Labs., Beckenham, England, BR3 3BS).

Both of the fluorescent techniques (Nairn, 1969; Phillips and Lewis, 1970), the fluorescent index used for semi-quantitation (Klein and Klein, 1964), and the Wild M20 dual illuminator microscope (Wild, Canada) which employs an interference (FITC 490 + OG/1) filter with a blue light source derived from a high intensity halogen lamp (Iodine Quartz), have all been described previously (Lewis and Phillips, 1972). Cells giving a red appearance with discrete apple-green fluorescent point staining were regarded as positive, and unstained cells visualized as red spheres were negative. Diffusely staining dead cells were excluded. Under these conditions it was possible to identify and further exclude small lymphocytes because of their size, and plasma cells because of the characteristic nuclear/cytoplasmic morphology. In all instances histological sections, electron micrographs and cytological smears stained conventionally were prepared and examined in addition. The serum of 26 patients was also investigated following either intradermal autoimmunization with irradiated tumour cells alone (Ikonopisov *et al.*, 1970), or in combination with oral BCG administration (Lewis and Raymond, 1975). In addition, tumour and sera from 10 patients who had been treated with intravenous phytohaemagglutinin alone (Lewis *et al.*, 1971), or in combination with autoimmunisation were examined. Finally, a further 9 malignant melanoma cases were studied in a similar fashion, and the positive direct fluorescent staining small lymphocytes and plasma cells identified and counted.

### RESULTS

The fluorescent indices obtained on direct immunofluorescence are compared with those obtained by indirect immunofluorescence on the 37 melanoma patients (see Fig. 1); the dark areas represent the fluorescent index on the addition of anti-human gammaglobulin conjugate

FIG. 1.—Anti-membrane antibody levels in two groups of melanoma patients (dotted areas) (measured by indirect immunofluorescence) with level of antibody on tumour cell surfaces (dark areas) measured by direct immunofluorescence.

TABLE I.—*Comparison Between Autoimmunization and Intravenous Phytohaemagglutinin Production of Serum Antibody and Tumour-bound Antibody in Melanoma Patients*

| | Serum Ab measured by indirect I.F. | | Tumour-bound Ab measured by direct I.F. | |
|---|---|---|---|---|
| Immunizing procedure | Positive | Negative | Positive | Negative |
| Autoimmunization with or without subsequent BCG | 17 | 9 | 2 | 24 |
| Intravenous PHA with or without autoimmunization | 7 | 3 | 9 | 1 |

alone (direct) and the dotted areas that after the administration of serum from the same individual patients (indirect). The fluorescent index of the tumour cells prior to administration of the serum bears no relationship to the index recorded when serum is added, irrespective of whether anti-tumour antibody was present in the serum or not. In previous reported studies we showed that an index of 0·3 or less gave a weak or unrecordable intensity of fluorescence using a semiquantitative comparator system and a quantitative immunofluorescence microscope (Lewis *et al.*, 1973*a*). In none of the 37 cases examined does the index exceed this figure and in many was less than 0·10 (Fig. 1). In 24 of the 26 autoimmunized cases the index was lower than 0·3 on direct fluorescence testing, while in 17 of the 26 cases the index was greater than 0·3 on indirect immunofluorescent testing with serum taken after immunization (see Table I). Of the 10 cases who were treated with intravenous phytohaemagglutinin, 9 had significant numbers of stained tumour cells on direct immunofluorescence, implying that when antibody is present in tumour cells it can be detected using our methods.

On comparing the fluorescent index of tumour cells to that of the host cells using direct immunofluorescence, in 9 cases the index of tumour cells again never exceeded 0·3, while in 4 instances it was above 0·3 for the host cells (Table II). Figure 2 shows examples of host cells with positive immunofluorescence on application of FITC or peroxidase-conjugated anti-human gamma globulin.

TABLE II.—*Comparison Between Immunoglobulin on Surface of Tumour Cells and Host Cells after Application of Fluorescent Conjugated Anti-human Gammaglobulin in Cell Suspensions from Nine Melanomata*

| Patient | Tumour cell Fluorescence index (direct I.F.) | Host cell Fluorescence index (direct I.F.) |
|---|---|---|
| PYK | 0·01 | 0·65 |
| SZ | 0·01 | 0·10 |
| KT | 0·01 | 0·10 |
| JP | 0·00 | 0·05 |
| PA | 0·05 | 0·20 |
| PE | 0·30 | 0·40 |
| CH | 0·18 | 0·30 |
| TH | 0·10 | 0·80 |
| FL | 0·00 | 0·25 |

Much of this immunoglobulin is likely to be unrelated to tumour antibody and merely represents normal lymphoid cells in the area of the tumour. It would, however, still give positive gammaglobulin on elution of such a tumour–host cell mixture. This line of investigation is being extended to include a more detailed morphological and functional examination of the host cell types and will be the subject of a further communication.

### DISCUSSION

Although there have been several publications showing the presence of anti-tumour antibody within tumours (Witz, 1973; Romsdahl and Cox, 1971; Ran and Witz, 1970), few of these reports have demonstrated that the antibody was attached to the surface of the tumour cells *per se*. In those instances where this was definitively shown to be true, the tumours studied were either lymphoid in origin (Klein *et al.*, 1969; Witz, Klein

FIG. 2.—Examples of host cells in tumour showing positive immunoglobulin with application of fluorescein-conjugated anti-human gammaglobulin or peroxidase-labelled anti-human gammaglobulin.

(a) Lymphocytes: demonstrating " polar capping " × 2000.
(b) Plasma cell with intracytoplasmic immunoglobulin in a fixed cell smear × 1200.
(c) Macrophage with immunoglobulin and engulfed particles × 1200.
(d) Negative tumour cell and adjacent lymphocyte shows positive reaction with peroxidase-labelled anti-human gammaglobulin × 2000.

and Pressman, 1969; Nishioka, 1971), or in the case of solid tumours, lymph node metastases (Morton, Eilber and Malmgren, 1971) or malignant effusions (Sjögren *et al.*, 1972; Witz, 1973; Ghose *et al.*, 1972). In all such cases, serum antibody might be expected to have reasonable access to the tumour cells. There is no reason to suppose that this would necessarily hold for the more common situation of solid tumour deposits lying outside the lymphatic or blood vascular systems.

The concept that a tumour will act as a sponge, soaking up antibody from the circulation, has been supported by the finding that removal of most of the total tumour mass in man (Morton *et al.*, 1971; Pilch and Riggins, 1966) and in animals (Witz, 1973), leads to a rise in antibody titres. An equally strong argument can be mounted, however, without considering the possibility of antibody reaching the tumour at all, namely that tumours release soluble antigen into the circulation and that this could be expected to complex with any free antibody already present. Removal of a tumour would naturally cause a dramatic drop in the levels of circulating antigen and antibody would become detectable; indeed, this has been demonstrated experimentally with a rat sarcoma system by Thomson *et al.* (1973*a*). Neither of these concepts constitutes a complete explanation, as we have shown previously (Lewis *et al.*, 1973*b*) and more recently Bodurtha *et al.* (1975), that the presence or absence of circulating antibody in melanoma patients cannot be related to the tumour burden at the time.

It has been suggested by Hall (1969) that immunoglobulin has an important role in aiding the maintenance of the osmotic equilibrium of the blood and as such would not be expected to leave the circulation under normal conditions where the vascular permeability has not been deranged. It is known, however, that tumour vessels are not normal, particularly in relation to their relative lack of elastica when compared to normal vessels. Nevertheless, as suggested by Hall (1969) the concept that certain forms of cellular immunity are required in order to carry antibody to sites of antigenic challenge outside the vasculature is attractive. In the case of dental cysts which contain high levels of immunoglobulin in the lumen, this has been related to migration of plasma cells through the cyst walls (Toller and Holborow, 1969). In keeping with this report are the further reports that IgM, IgA and IgG were identified in the plasma cells and small lymphocytes infiltrating the tumour substance of bladder carcinomata (Johansson and Ljungqvist, 1974), while in the case of breast carcinoma the amount of immunoglobulin eluted from the tumours, was related quantitatively to the number of plasma cells present within them (Roberts *et al.*, 1973).

Our preliminary investigations of immunoglobulin on host cells support the above reports. Further, our finding of an extremely low level of positively staining tumour cells by direct immunofluorescence confirm those of Kopf, Silberberg and Cooper (1966), who failed to detect antibody on the surface of malignant melanoma, and Thomson, Steele and Alexander (1973*b*), who found no immunoglobulin on the surface of rat sarcoma cells.

Xenogeneic labelled anti-tumour antisera injected into the circulation have been shown to localize in the tumour against which it was raised (Ghose *et al.*, 1972). Pressman (1966) noted that this localization occurred in the vascular bed of the target tissue. In other reports, there is a definite lag period before localization occurs, ranging from 36 h to more than 2 days. These findings would not support the tumour sponge concept, and it is tempting to speculate that host cells might be responsible for carrying antibody into the tumour. In recent, more definitive experiments on the rat sarcoma model, no immunoglobulin is detected on the tumour cells of both lymphocyte and macrophage series following sedimentation velocity separation of single cell tumour suspensions (Haskill, Proctor and Yamamura, 1975).

More importantly, results reported in the present study in which tumour cells were found to be heavily coated with immunoglobulin following injection of phytohaemagglutinin to the patient, serve to illustrate that if immunoglobulin is present in reasonable quantities on the tumour cell surface, it is readily identifiable using the techniques we have described. We do not have a ready explanation for why this occurred and the study was stimulated originally by the observation that in the case of the single patient treated with intravenous phytohaemagglutinin a dramatic regression of multiple subcutaneous tumour nodules occurred (Lewis *et al.*, 1971). The tumour cells within these nodules were found to be heavily coated with immunoglobulin while in the case of those removed prior to administration of PHA there was no significant staining of tumour cells on direct fluorescence irrespective of whether antibody could be detected in the serum or not.

While the question as to exactly where immunoglobulin is located within extravascular tumour deposits must remain incompletely answered, it is clear that (in the presence of serum anti-tumour antibodies) except under special circumstances, immunoglobulin would not appear to be bound to the malignant melanoma tumour cell surface and that it is present on host cells. Certainly, the concept that antibody can act as a blocking agent to cellular immunity at the target cell level would seem to be unlikely, though complexing of free antibody with antigen within tumours could still constitute a valid mechanism for abrogation of cell-mediated immune responses within tumours.

The authors would like to express their thanks to many clinical colleagues for providing material for their study, and for the expert technical assistance of Mr Donald Hay, Miss H. Lyons, Miss C. Quirk, Mr E. Hedderson and Mrs M. Watson.

This work was supported by the National Cancer Institute of Canada.

### REFERENCES

BANSAL, S. C. & SJÖGREN, H. O. (1971) Unblocking Serum Activity *in vitro* in the Polyoma System may Correlate with Antitumor Effects of Antiserum *in vivo*. *Nature, New Biol.*, **233**, 76.

BODURTHA, A. J., CHEE, D. O., LAUCIUS, J. F., MASTRANGELO, M. J. & PREHN, R. T. (1975) Clinical and Immunological Significance of Human Melanoma Cytotoxic Antibody. *Cancer Res.*, **35**, 189.

GHOSE, T., NORVELL, S. T., GUCLU, A., CAMERON, D., BODURTHA, A. & MACDONALD, A. S. (1972) Immunochemotherapy of Cancer with Chlorambucil Carrying Antibody. *Br. med. J.*, iii, 495.

HALL, J. G. (1969) Hypothesis: Effector Mechanisms in Immunity. *Lancet*, i, 25.

HASKILL, J. S., PROCTOR, J. W. & YAMAMURA, Y. (1975) Host Responses within Solid Tumors. I. Monocytic Cells within Rat Sarcomas. *J. natn. Cancer Inst.*, **54**, 387.

HELLSTRÖM, I., SJÖGREN, H. O., WARNER, G. & HELLSTRÖM, K. E. (1971) Blocking of Cell-mediated Tumor Immunity by Sera from Patients with Growing Neoplasms. *Int. J. Cancer*, **7**, 226.

IKONOPISOV, R. L., LEWIS, M. G., HUNTER-CRAIG, I. D., BODENHAM, D. C., PHILLIPS, T. M., COOLING, C. I., PROCTOR, J., FAIRLEY, G. H. & ALEXANDER, P. (1970) Auto-immunisation with Irradiated Tumour Cells in Human Malignant Melanoma. *Br. med. J.*, ii, 752.

JOHANSSON, B. & LJUNGQVIST, A. (1974) Localisation of Immunoglobulins in Urinary Bladder Tumors. *Acta path. microbiol. Scand.*, (A) **82**, 559.

KLEIN, G., CLIFFORD, P., HENLE, G., HENLE, W., GEERING, G. & OLD, L. J. (1969) EBV-associated Serological Patterns in a Burkitt Lymphoma Patient during Regression and Recurrence. *Int. J. Cancer*, **4**, 416.

KLEIN, E. & KLEIN, G. (1964) Antigenic Properties of Lymphomas Induced by the Moloney Agent. *J. natn. Cancer Inst.*, **32**, 547.

KOPF, A. W., SILBERBERG, I. & COOPER, N. S. (1966) Immunohistochemical Study of Human Malignant Melanoma for the Presence of Gamma Globulin. *J. invest. Derm.*, **47**, 83.

LEWIS, M. G., AVIS, P. J. G., PHILLIPS, T. M. & SHEIKH, K. (1973*b*) Tumor-associated Antigens in Human Malignant Melanoma. *Yale J. Biol. Med.*, **46**, 661.

LEWIS, M. G., HUMBLE, J. G., LEE, S. E. & PHILLIPS, T. M. (1971) The Effects of Intravenous Phytohaemagglutinin in a Patient with Disseminated Malignant Melanoma: A Clinical and Immunological Study. *Revue Eur. Étud. Clin. Biol.*, **16**, 924.

LEWIS, M. G., IKONOPISOV, R. L., NAIRN, R. C., PHILLIPS, T. M., FAIRLEY, G. H., BODENHAM, D. C. & ALEXANDER, P. (1969) Tumour Specific

Antibodies in Human Malignant Melanoma and their Relationship to Extent of the Disease. *Br. med. J.*, iii, 547.

LEWIS, M. G., JERRY, L. M. & SHIBATA, H. (1975) Prospective of Specific Immunotherapy. Proc. XI Cancer Cong. Florence, 20–26 October, 1974. *Excerpta Medica.* (In the press.)

LEWIS, M. G., MCCLOY, E. & BLAKE, J. (1973*a*) The Significance of Humoral Antibodies in the Localisation of Human Malignant Melanoma. *Br. J. Surg.*, **60**, 443.

LEWIS, M. G. & PHILLIPS, T. M. (1972) The Specificity of Surface Membrane Immunofluorescence in Human Malignant Melanoma. *Int. J. Cancer*, **10**, 105.

LEWIS, M. G. & RAYMOND, M. (1975) Humoral and Cellular Host Reactions to Melanoma Antigens. *Behring Inst. Mitt.*, **56**, 120.

LITTLE, J. H. (1972) In *Proc. International Cancer Conference, Sydney, Australia.* Melanoma and Skin Cancer, p. 107. V. C. N. Bight, Government Printer.

MASTRANGELO, M. J., LAUCIUS, J. F. & OUTZEN, H. C. (1974) Fundamental Concepts in Tumor Immunology: A Brief Review. In *Seminars in Oncology*, **1**, 291.

MORTON, D. L., EILBER, F. R. & MALMGREN, R. A. (1971) Immune Factors in Human Cancer: Malignant Melanomas, Skeletal and Soft Tissue Sarcomas. *Prog. exp. Tumor Res.*, **14**, 25.

NAIRN, R. C. (1969) *Fluorescent Protein Tracing*, 3rd ed. Edinburgh: E. & S. Livingstone, Ltd.

NAIRN, R. C. (1974) In *Immunological Aspects of Skin Diseases: Malignant Melanoma*, Ed. L. Fry and P. P. Seah. pp. 153–191. Lancaster: Medical and Technical Publishing Co. Ltd.

NISHIOKA, K. (1971) Complement and Tumor Immunology. *Adv. Cancer Res.*, **14**, 231.

PHILLIPS, T. M. & LEWIS, M. G. (1970) A System of Immunofluorescence in the Study of Tumour Cells. *Revue Eur. Étud. Clin. Biol.*, **15**, 1016.

PILCH, Y. H. & RIGGINS, R. S. (1966) Antibodies to Spontaneous and Methylcholanthrene-induced Tumors in Inbred Mice. *Cancer Res.*, **26**, 871.

PRESSMAN, D. (1966) *In vivo* Localisation of Antitumor Antibodies. *Gann Monogr.*, **4**, 149.

RAN, M. & WITZ, I. P. (1970) Tumor-associated Immunoglobulins. The Elution of IgG2 from Mouse Tumors. *Int. J. Cancer*, **6**, 361.

RITCHERS, A. & KASPERSKY, C. L. (1975) Surface Immunoglobulin-positive Lymphocytes in Human Breast Cancer Tissue and Homolateral Axillary Lymph Nodes. *Cancer, N.Y.*, **35**, 129.

ROBERTS, M. M., BASS, E. M., WALLACE, I. W. J. & STEVENSON, A. (1973) Local Immunoglobulin Production in Breast Cancer. *Br. J. Cancer*, **27**, 269.

ROMSDAHL, M. M. & COX, I. S. (1971) Evidence for Enhancing Antibodies in Human Sarcomas. *Proc. Am. Ass. Cancer Res.*, **12**, 66.

SARMA, K. P. (1970) The Role of Lymphoid Reaction in Bladder Cancer. *J. Urol.*, **104**, 843.

SJÖGREN, H. O., HELLSTRÖM, I., BANSAL, S. C., WARNER, G. A. & HELLSTRÖM, K. E. (1972) Elution of "Blocking Factor" from Human Tumors Capable of Abrogating Tumor Cell Destruction by Specifically Immune Lymphocytes. *Int. J. Cancer*, **9**, 274.

THOMSON, D. M., SELLENS, V., ECCLES, S. & ALEXANDER, P. (1973*a*) Radioimmunoassay of Tumour Specific Transplantation Antigen of a Chemically Induced Rat Sarcoma: Circulating Soluble Tumour Antigen in Tumour Bearers. *Br. J. Cancer*, **28**, 377.

THOMSON, D. M., STEELE, K. & ALEXANDER, P. (1973*b*). The Presence of tumour-specific Membrane Antigen in the Serum of Rats with Chemically Induced Sarcomata. *Br. J. Cancer*, **27**, 27.

TOLLER, P. A. & HOLBOROW, E. J. (1969) Immunoglobulins and Immunoglobulin-containing Cells in Cysts of the Jaws. *Lancet*, ii, 178.

WITZ, I. P. (1973) The Biological Significance of Tumor-bound Immunoglobulins. *Curr. Top. Microbiol. Immunol.*, **61**, 151.

WITZ, I., KLEIN, G. & PRESSMAN, D. (1969) Moloney Lymphoma Antibodies from Mice; Localisation in Spleens of Moloney Lymphoma Bearing Mice. *Proc. Soc. exp. Biol. Med.*, **130**, 1102.